# Orthostatic Change in Blood Pressure and Incidence of Atrial Fibrillation: Results from a Bi-Ethnic Population Based Study

**DOI:** 10.1371/journal.pone.0079030

**Published:** 2013-11-11

**Authors:** Sunil K. Agarwal, Alvaro Alonso, Seamus P. Whelton, Elsayed Z. Soliman, Kathryn M. Rose, Alanna M. Chamberlain, Ross J. Simpson, Josef Coresh, Gerardo Heiss

**Affiliations:** 1 Department of Epidemiology and Medicine, Johns Hopkins University, Baltimore, Maryland, United States of America; 2 Division of Epidemiology and Community Health, University of Minnesota, Minneapolis, Minnesota, United States of America; 3 Department of Epidemiology and Prevention EpiCare, Wake Forest University School of Medicine, Winston-Salem, North Carolina, United States of America; 4 Department of Epidemiology and Medicine, University of North Carolina, Chapel Hill, North Carolina, United States of America; 5 Department of Health Sciences Research, Mayo Clinic, Rochester, Minnesota, United States of America; University of Otago, New Zealand

## Abstract

**Background:**

Autonomic fluctuations are associated with the initiation and possibly maintenance of atrial fibrillation (AF). However, little is known about the relationship between orthostatic blood pressure change, a common manifestation of autonomic dysfunction, and incident AF.

**Methods:**

We examined whether supine-to-standing changes in systolic blood pressure (SBP) are associated with incident AF in 12,071 African American and white men and women aged 45–64 years, enrolled in the Atherosclerosis Risks in Communities (ARIC) study. Orthostatic hypotension (OH) was defined as a supine-standing drop in SBP by ≥20 mmHg or diastolic blood pressure by ≥10 mmHg. AF cases were identified based on study scheduled 12-lead ECG, hospital discharge ICD codes, and death certificates through 2009.

**Results:**

OH was seen in 603 (5%) at baseline. During an average follow-up of 18.1 years, 1438 (11.9%) study participants developed AF. Incident AF occurred more commonly among those with OH than those without, a rate of 9.3 vs. 6.3 per 1000 person years, (p<0.001). The age, gender, and race adjusted hazard ratio (95%CI) of AF among those with OH compared to those without was 1.62 (1.34, 2.14). This association was attenuated after adjustment for common AF risk factors to HR 1.40 (1.15, 1.71), a strength similar to that of diabetes or hypertension with AF in the same model. A non-linear relationship between orthostatic change in SBP and incident AF was present after multivariable adjustment.

**Conclusions:**

OH is associated with higher AF incidence. Whether interventions that decrease OH can reduce AF risk remains unknown.

## Introduction

Atrial fibrillation (AF) affects approximately 3 million North Americans and the prevalence is projected to double by 2050. [1], [2] AF shares common risk factors with other cardiovascular diseases (CVD) including age, obesity, hypertension, diabetes, cigarette smoking, and preexisting heart disease. [3], [4] However, not all individuals who develop AF have CVD risk factors, and in younger individuals as many as 45% may not have underlying CVD. [5] Furthermore, both divisions branches of the autonomic nervous system (ANS) i.e., sympathetic and parasympathetic may be strong modulators of the AF substrate, leading to the initiation and/or maintenance of AF [6]. Thus, constructs of neuro-humoral system dys-regulation may provide additional, patho-physiological insights and also help assist in identifying individuals at risk for developing AF.

The ANS plays an important role in complex mechanisms involved in maintaining blood pressure. During a change from supine or seated to standing position, an initial withdrawal of parasympathetic tone followed by an increase in sympathetic tone in response to the redistribution of blood in the capacitance vessels of the lower limbs is seen [7]. The measurement of orthostatic blood pressure change is an easy, inexpensive, and relatively quick procedure that can provide some information about an individual’s ANS dys-regulation [8]. Prospective studies report that those with a substantial drop in their blood pressure upon standing categorized as orthostatic hypotension (OH) have reportedly a two-fold higher stroke risk, [9] and a 50–100% increased mortality rate than those without OH. [10], [11] However, there is limited information from population based studies to describe the association between OH and AF incidence. [12] We hypothesize that individuals who have either a drop or an increase in blood pressure upon standing may be at an increased risk of AF.

## Methods

### Study Sample

The ARIC Study enrolled 15,792 men and women ages 45–64 years sampled from four U.S. communities using area-probability sampling from Washington County, Minneapolis, Jackson, and Winston Salem. The ARIC study has been approved by the Institutional Review Boards (IRB) of all participating institutions, including the IRBs of the University of Minnesota, Johns Hopkins University, University of North Carolina, University of Mississippi Medical Center, and Wake Forest University. All participants gave written informed consent in each one of the study visits. Baseline examinations of the cohort were conducted from 1987 to 1989 to collect standardized information on socioeconomic indicators, medical history, family history, CVD risk factors, serum chemistries, electrocardiograms (ECGs), medication use, and anthropometrics. Three follow up examinations (visits) followed the baseline visit, as well as annual telephone interviews and active surveillance of hospitalizations and death. A complete description of the ARIC communities and of the design has been published elsewhere. [13].

For this analysis, participants were excluded if they were either missing or had poor quality data on postural change in blood pressure measured at study baseline (n = 2555), prevalent AF or atrial flutter identified from ECGs at baseline (n = 37), other abnormalities in heart rhythm (advanced AV blocks, pacemakers, wandering atrial beats, supra-ventricular or ventricular tachycardia; n = 242), self-identified race as neither African American nor Caucasian (n = 48), were African Americans from Minnesota or Washington county (n = 55), or were missing important covariates (n = 1238). Data from the remaining 12,071 study participants were included in the analyses.

### Ascertainment of AF

Electrocardiograms (ECGs) during the baseline visit were used to exclude individuals with prevalent AF or atrial flutter. Incident AF cases through December 31, 2009 were identified from 3 sources: 1) study scheduled ECGs that were recorded triennially through 1998, 2) hospital discharge records using ICD codes through 2009, and 3) death certificates through 2009.

ECGs were recorded using MAC PC Personal Cardiographs (Marquette Electronics, Inc., Milwaukee, WI) and were then transmitted to the ARIC ECG Reading Center for computer coding and verified by trained experts to confirm the diagnosis of AF. [14].

Annual follow-up telephone calls made to cohort participants identified any hospitalizations or vital status. In addition, local hospitals records and the National Death Index were surveyed to ascertain cardiovascular events or death. Hospital discharge records were abstracted from all participant hospitalizations and AF was identified as an ICD-9 discharge code of 427.31 or 427.32 for atrial flutter at any position. AF was also identified by the presence of ICD-9 code 427.3 or ICD-10 code I48 at any position in the death certificate. ^14^ AF occurring simultaneously with heart revascularization surgery or other cardiac surgery involving heart valves or septa was not considered an incident event and follow-up was continued beyond that surgical AF related event. More than 99% of the AF cases were identified at hospitalization. Within the ARIC cohort, the sensitivity and specificity of a hospital discharge diagnosis for AF was found to be 84% and 98%, respectively. [15].

### Orthostatic Changes in Blood Pressure

At the baseline examination, after 20 minutes of resting in the supine position, blood pressure was measured in the study participants approximately every 30 seconds for 2 min (2–5 measurements, 90% had ≥4 measurements) using Dinamap 1846 SX, automated oscillometric device. Participants were then asked to stand, and BP measurements were repeated during the first 2 min after standing (2–5 measurements, 91% had ≥4 measurements). BP change was defined as the difference between the average standing and average supine after excluding the 1st standing measurement, as BP homeostasis occurs during the first 30 s after standing [16]. Postural hypotension was defined as a systolic blood pressure (SBP) drop of ≥20 mmHg or diastolic blood pressure (DBP) of ≥10 mmHg when changing position from supine to standing.

### Other Covariates

Race, education attainment, cigarette smoking status, pack-years of smoking, and alcohol drinking status were determined by self-report. Body mass index (BMI) was calculated as weight (in kilograms) divided by height (in meters) squared. Diabetes was defined as fasting glucose ≥126 mg/dL, non-fasting glucose of ≥200 mg/dL, self-report of a physician diagnosis or current use of glucose lowering medications. Participants’ sitting blood pressure was measured at baseline 3 times, with the mean of the last 2 measurements used. Hypertension, defined as SBP of ≥140 mmHg, DBP≥90 mmHg, or the use of a blood pressure medication in the past 2 weeks. Prevalent CHD was defined as the presence of a self-report of myocardial infarction (MI), coronary artery bypass, angioplasty, or MI based on the baseline ECG. Prevalent Heart Failure (HF) was identified by the Gothenburg criteria or self-report use of HF medication use in the past 2 weeks. [17] Medication history was obtained by self-report of medication intake during last two weeks and by reviewing medication brought by participants to their visit. Each medication was coded by trained and certified interviewers with the use of a computerized medication classification system. Medications in the classes of anti-hypertensive, anti-depressants, anti-psychotics, anti-cholinergics, sedatives, narcotics, and nitro compounds were grouped together as medication with potential to induce orthostatic hypotension.

### Statistical Methods

The analysis was performed using SAS version 9.1.3 (SAS Institute, Cary, NC). Cumulative AF proportions during follow up time were estimated using Kaplan Meier methods, and a graph with 1- survival (t) by OH status was generated. Cox proportional hazards models were used to estimate multivariable adjusted hazard ratios of AF by OH status with varying degrees of adjustment for potential confounders. We examined the possibility of a non-linear relationship between orthostatic changes in SBP and AF incidence using restricted cubic splines for orthostatic BP change as a continuous variable with no change as reference. [18] A partial likelihood ratio test using models with only linear terms was compared to a model containing both linear and the cubic spline terms, which suggested a non-linear association with increased risk (though statistically non-significant) in those with increase in SBP upon standing. Therefore, in addition to focusing on OH which is an easily available clinical measure, we estimated risk of AF at either ends of orthostatic SBP change while considering those between the 5^th^ and 95^th^ percentile as the referent. Also, we also examined its association with between OH and AF stratified by several risk groups including race, gender, diabetes, hypertension, intake of medications that may predispose to OH, and history of CHD. Analysis restricted to those without diabetes, hypertension, heart failure, and CHD were also conducted. The proportional hazards assumption was checked for OH and there was no gross violation (p value for time interaction term = 0.34). A p value of <0.05 on a two tailed test was considered significant for the analysis and a p<0.1 was considered significant for interactions.

## Results

**Figure 1:**
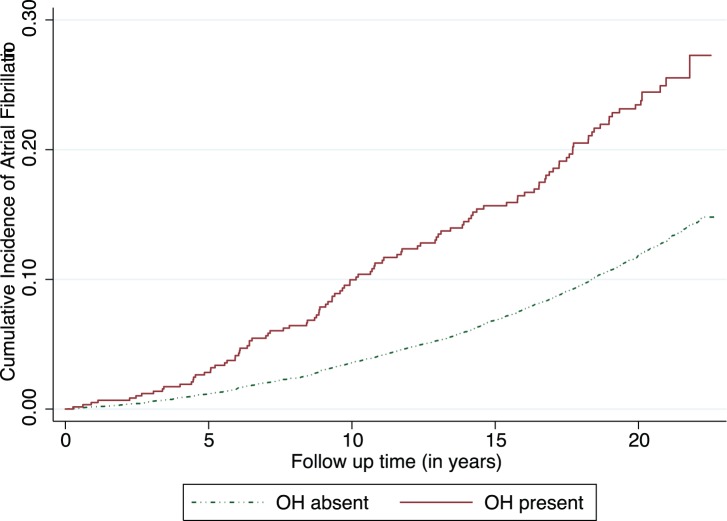
Orthostatic change in systolic blood pressure (standing-supine) at baseline visit and multivariable adjusted hazard ratio of atrial fibrillation: The Atherosclerosis Risks in Communities Study (1987–89 to 2009). In the upper panel, the line shows hazard ratio (HR) and the grey bands represents 95% confidence interval (95% CI) for hazard ratio with no change in blood pressure upon standing as reference. In the bottom panel, the line represents smoothed density plots showing distribution of the orthostatic change in systolic blood pressure (SBP) in the study sample. The extreme values of SBP change (0.25 percentile) at each extreme were removed before plotting above to remove the effect on restricted portion from outliers (though including them didn’t change shape much).

We analyzed data from 12,071 ARIC cohort participants of whom 55% were female and 25% African Americans. At baseline, 5% of participants had orthostatic hypotension, 34% had hypertension, 9% had diabetes, 5% had CHD, and 4% had HF. The distribution of orthostatic change in blood pressure on changing position from supine to standing is presented at the bottom of **Figure 1** **.** They were followed up for an average of 18 years with a cumulative follow up of 220,000 person-years. During this time 1,438 (11.9%) of the study participants developed AF. The differences in participant baseline characteristics are presented in **Table 1** stratified by incident AF. Those who developed AF were older, had a higher burden of traditional CVD risk factors, a higher prevalence of low formal education attainment, higher alcohol consumption, and were more likely to have orthostatic hypotension.

**Table 1:** Characteristics of the study sample (n = 12, 071) by incident AF status.

Characteristic	Incident AF (n = 1438)	No AF (n = 10633)
	Mean (SD) or proportion	Mean (SD) or proportion
**Age (years)**	56.75(5.5)	53.76 (5.7)
**Female**	45	56
**African American**	19	26
**Education**		
** Less than high school**	28	22
** >High and<college**	39	42
** College or professional**	32	37
**Hypertension**	46	32
**Diabetes**	14	9
**Prevalent CHD**	10	4
**Prevalent HF**	8	4
**Current cigarette smoker**	30	25
**Former cigarette smoker**	35	32
**Cigarette years of smoking**	440.7 (510.8)	306.4 (424.2)
**Alcohol intake in grams/week**	47.9 (100.7)	41.4 (93.9)
**Systolic blood pressure (mm of Hg)**	125.3 (20.0)	120.4 (18.7)
**Diastolic blood pressure (mm of Hg)**	73.5 (11.6)	73.3 (11.1)
**LDL-cholesterol (mg/dl)**	138.6 (38.4)	136.9 (38.9)
**HDL-cholesterol (mg/dl)**	48.3(16.0)	52.4 (17.2)
**Body mass index (Kg/mˆ2)**	28.6 (5.7)	27.4 (5.2)
**Cornell voltage (mV)**	1.28 (0.6)	1.20 (0.5)
**Mean change in systolic blood pressure**	−1.6 (12.0)	−0.3 (10.6)
**Mean change in diastolic blood pressure**	2.2 (6.1)	3.1 (5.6)
**Orthostatic hypotension**	0.08	0.05

Results from the Atherosclerosis Risk in Communities Study: 1987–89 through 2009.

AF = Atrial Fibrillation, CHD = Coronary Heart Disease, HF = Heart Failure.

All the characteristics are statistically different with p<0.05 between the incident AF and no AF groups.

### Orthostatic Hypotension and AF

**Figure 2:**
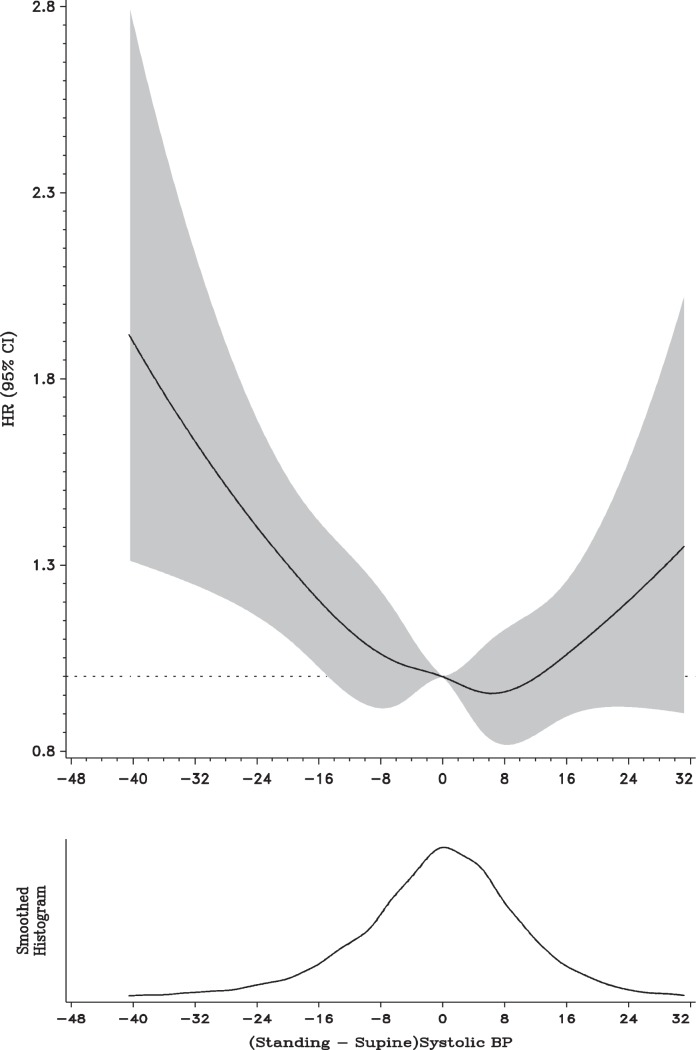
Cumulative incidence of atrial fibrillation by presence of orthostatic hypotension at baseline. Results from the Atherosclerosis Risks in Communities Study 1987–89 through 2009 (study n = 12, 071, incident atrial fibrillation n = 1438).

Incident AF occurred more commonly among those who had OH at baseline than those without (18.4% vs. 11.6%, p<0.001). The cumulative event proportion curve (1- survival at time t) graph showed higher rate of AF among those with OH throughout the study period (log-rank test p<0.001, **Figure 2**). The age adjusted incidence rate of AF was also higher among those with OH compared to those without OH 9.3 (95% CI: 7.7, 11.2) vs. 6.3 (95% CI: 5.9, 6.6 per 1000 person-years. The unadjusted hazard ratio of AF among those with orthostatic hypotension compared to those without was 2.22 (95% CI 1.80–2.65). Adjustment for age, gender, race, and study center attenuated the relation to 1.62 (95% CI 1.34–2.14), which was further attenuated to a HR 1.40 (95% CI 1.15–1.71) after additional adjustment with common AF risk factors including BMI, heart rate, SBP, DBP, anti-hypertensive medications intake, diabetes, smoking status, alcohol consumption, HDL-C, LDL-C, CHD, and heart (**Table 2**).

**Table 2:** Hazard Ratio of Atrial Fibrillation by Orthostatic Hypotension at Baseline.

Model #	Study sample	HR	95% CI
**Model 1**	All	2.22	(1.80,2.65)
**Model 2**	All	1.62	(1.34,2.14)
**Model 3**	All	1.40	(1.15,1.71)
**Model 3**	No CHD and no HF (92% of study sample)	1.29	(1.03,1.62)
**Model 3**	No CHD or HF or diabetes or hypertension (60.1% of study sample)	1.31	(0.93,1.86)

Results from the Atherosclerosis Risk in Communities Study: 1987–89 through 2009.

CHD = coronary heart disease, HF = heart failure, HR = hazard ratio, 95% CI = 95% confidence interval.

Model 1 is unadjusted.

Model 2 is adjusted for age, race, gender, and study center.

Model 3 is adjusted for model 2+ BMI, heart rate, systolic blood pressure, diastolic blood pressure, anti-hypertensive medications intake, diabetes, smoking status, alcohol consumption, HDL-C, LDL-C, CHD.

The strength of this association is similar to the association of diabetes (1.42; 95% CI: 1.21–1.66) or hypertension (1.38; 95% CI: 1.20–1.59) with AF in the same multivariable model that includes OH and other potential confounders. In additional analyses that excluded those with diabetes, hypertension, or CHD, associations persisted.

### Orthostatic Hypotension and AF by Subgroups

We also performed several stratified analysis to better assess the putative relationship by several sub-groups (**Table 3**). The association of OH with AF was stronger in women than in men.

**Table 3:** Risk of Atrial Fibrillation among those with Orthostatic Hypotension as compared to those without by several sub-groups.

Strata	Number (%of sample)	HR	95% CI	p value
**Overall**	12071 (100%)	1.4	(1.15,1.71)	–
**Age<55 years**	6384 (52.9)	1.44	(0.93,2.25)	0.74
**Age ≥55 years**	5687 (47.1)	1.51	(1.21,1.87)	
**Female**	6647(55.1)	1.66	(1.27,2.18)	0.07
**Male**	5425 (44.9)	1.20	(0.89,1.16)	
**African American**	3056 (25.3)	1.82	(1.20,2.70)	0.18
**Caucasian**	9015 (74.7)	1.32	(1.05,1.65)	
**Prevalent CHD**	564 (4.7)	1.35	(0.74,2.49)	0.47
**No CHD**	11507 (95.3)	1.41	(1.15,1.75)	
**Diabetes**	1116 (9.2)	1.34	(0.82,2.18)	0.29
**No Diabetes**	10955 (90.8)	1.43	(1.15,1.78)	
**Hypertension**	4064 (33.7)	1.44	(1.12,1.85)	0.71
**No hypertension**	8007 (6.3)	1.33	(0.96,1.85)	
**Medication intake**	4098 (34.0)	1.66	(1.26,2.19)	0.03
**No medications intake**	7973 (66.1)	1.08	(0.76,1.54)	

Results from the Atherosclerosis Risk in Communities Study: 1987–89 through 2009.

CHD = Coronary Heart Disease, HF = Heart Failure, HR = Hazard Ratio.

p for interaction will be low if the relationship is different in the two strata of variable, and refers to p value for the term Orthostatic Hypotension*Strata variable in a model including both these variables and other potential confounders.

Medications refers to the drugs that have tendency to cause orthostatic hypotension including anti-hypertensive.

Similarly, individuals taking medications that may increase the risk of OH showed a stronger association between OH and AF, whereas no association was seen among those not taking these medications (HR (95% CI) of 1.66 (1.26, 2.19) vs. 1.08 (0.76, 1.54); p for interaction = 0.03).

There was no significant difference in the association of OH with AF risk between the other sub-groups including no differences between whites and blacks.

### SBP Change upon Standing and AF

The relationship of continuous change in SBP with AF was non-linear (**Figure 1**). We found an asymmetrical reversed J-shaped relationship with the SBP changes at either extreme showing a higher risk of AF as compared to the majority of the sample in the middle. This relationship retained its shape after multivariable adjustment but those with an increase in SBP were not significantly different than those with no change. As compared to those with SBP change within 5^th^ to 95^th^ centile (referent group), those with SBP decrease (>19 mm of Hg or more) had a higher risk of AF with a HR of 1.58 (95% CI: 1.30–1.91). However, compared to the same referent those with SBP increase (>16 mm of Hg or more) did not have significantly higher risk of AF with a HR of 1.13 (95% CI: 0.90–1.41. Diastolic BP change when standing from supine was more narrowly distributed than systolic BP change. Though its association with AF was similar to systolic BP; it was not statistically significant (data not shown).

## Discussion

In this prospective analysis of a large population based cohort, we found a 40% higher risk of AF among those with OH, compared to those without, independent of several potential confounders. The strength of this association is similar to the association of diabetes or hypertension with AF in the multivariable model that includes OH and other potential confounders. The association was also found to be non-linear.

While both experimental and human studies point to a role of autonomic perturbations and modulations in initiation, maintenance, and treatment of AF [19], [20] [21], only one other population based study has examined the association between OH and AF [12]. Contrary to the previous study, we found a similar association among those with and without hypertension, and similar to that study the association showed about 30–40% increased risk, and we have extended the study by exploring the relationship over the range of SBP change [12]. Notably, the estimated effect size is likely smaller than the true value as the reliability of single measurement is moderate, [22] and the follow up is lengthy in both studies. There was no association observed in the subgroup that did not take orthostatic inducing medications. One can only contemplate that those with OH in this group may be less exposed to its effect than those on medications. Importantly, this association persisted with similar strength in those without prevalent HF or CHD or hypertension or diabetes (60% of the cohort), although it was statistically non-significant.

Interestingly, we noted a small signal of increased risk for AF in participants who had a rise in SBP upon standing (though statistically non-significant in multivariable model as compared to those without change). Also, a U shaped relationship between orthostatic change in blood pressure and silent cerebrovascular disease has been reported [12]. Based on our results it is hard to say that the non-significant signal seen at this tail of SBP change is a true effect and would need examination in other studies.

A sustained decrease or increase in blood pressure upon standing may reflect among other causes, an inability of the ANS to come to a balance quickly. Interestingly, both renal artery denervation (sympathetic) [23] or wide circumferential ablation (parasympathetic) [24], when done in addition to pulmonary vein isolation, has been associated with a lower recurrence of AF in those with paroxysmal/refractory AF, suggesting that an imbalance may lead to fluctuations in ANS leading to initiation of AF and that such wide fluctuations may be reflected in the orthostatic change in blood pressure. Understanding of interventions that may improve OH, except in the settings of severe autonomic dysfunction, is limited. A deeper understanding of these interventions as well as whether they improve long term cardiovascular outcomes may benefit a large proportion of the general population.

Strengths of this study include being a community based sample of biracial middle aged men and women with long term follow up and a characterization of important potential confounders at baseline. There are several limitations such as, a) the moderate reliability of orthostatic blood pressure change reading at one visit only and its potential misclassification over time, which may drive the effect estimate towards null; b) we will miss those with paroxysmal AF or managed in outpatient settings and who did not have a hospital admission through the end of follow up – this is unlikely to differ by OH status and thus we do not expect a large bias from this. Also, it is unlikely that these free dwelling community participants were meaningfully dehydrated during a field center visit. Measures of peripheral neuropathy were not available. Lastly, cardiac structural and functional measures such as left ventricular mass, left ventricular ejection fraction, or pulmonary artery pressures were not available and may act as potential confounders.

In conclusion, we found that orthostatic hypotension, a simple clinical measurement, is associated with an elevated AF risk by 40% during an average follow up of 18 years. The strength of this a relationship is similar to that of diabetes or hypertension with AF. Future studies examining mechanisms linking orthostatic change in BP with AF including a closer look at autonomic function by measures such as heart rate variability may improve our understanding. Though causal association is difficult to prove, research exploring whether interventions to prevent and treat OH may improve cardiovascular outcomes merit attention.

## Acknowledgments

The authors thank the ARIC participants, staff, and investigators for their long-term contributions to ARIC.
